# Cross-cohort single-nucleus transcriptomic identification of a MEOX1+FAP+ACTA2-low extracellular matrix-remodeling fibroblast state in dilated cardiomyopathy

**DOI:** 10.3389/fgene.2026.1987893

**Published:** 2026-10-19

**Authors:** Yimeng Li

**Affiliations:** 1 Department of Cardiovascular Medicine, Liyang People’s Hospital, Liyang, Jiangsu, China; 2 Wuxi School of Medicine, Jiangnan University, Wuxi, Jiangsu, China

**Keywords:** cardiac fibroblast, DDAH1, dilated cardiomyopathy, extracellular matrix, FAP, MEOX1, single-nucleus RNA sequencing

## Abstract

**Background:**

Cardiac fibrosis is a major determinant of adverse remodeling in dilated cardiomyopathy (DCM), but the human disease-associated fibroblast state is incompletely resolved. We tested whether a non-classical MEOX1+FAP + ACTA2-low extracellular matrix (ECM)-remodeling state could be reproduced across independent human cardiac single-nucleus RNA-sequencing cohorts.

**Methods:**

Public GSE183852 data (25 non-failing donor and 13 DCM hearts) were used for discovery, and SCP1303 (16 normal and 11 DCM hearts) for external validation. Fibroblasts were independently reclustered. All inferential analyses used donors as the statistical unit. State abundance was assessed with Wilcoxon tests, 10,000-iteration donor bootstrap confidence intervals, leave-one-donor-out analyses, and heteroscedasticity-robust covariate models. Raw fibroblast counts were aggregated by donor for pseudobulk differential expression with age-tertile and sex adjustment. ECM activity was compared using pseudobulk and cell-derived donor summaries.

**Results:**

Discovery cluster two showed a MEOX1+FAP + ACTA2-low profile distinct from an ACTA2-high state and was enriched in DCM. Its mean abundance difference was 0.165 of fibroblasts (bootstrap 95% confidence interval 0.035–0.349), with a positive effect after omission of every donor. Age-tertile and sex adjustment preserved the effect magnitude, although the robust confidence interval marginally crossed zero. In SCP1303, the corresponding activated-fibroblast state was enriched in DCM (mean difference 0.192, bootstrap 95% confidence interval 0.144–0.256) and remained associated after sex adjustment. Donor-pseudobulk models identified higher DDAH1, MEOX1 and FAP expression in DCM after false-discovery-rate correction. Two ECM summaries were strongly correlated (Spearman *ρ* = 0.891), although group significance was method dependent.

**Conclusion:**

A reproducible MEOX1+FAP+, relatively ACTA2-low fibroblast state is associated with ECM remodeling in the analyzed human DCM cohorts. DDAH1 is a prioritized state-associated candidate, not an established causal regulator. Spatial and perturbation studies are required for mechanistic validation.

## Introduction

Dilated cardiomyopathy (DCM) is a major cause of heart failure and transplantation. Extracellular-matrix remodeling and fibrosis contribute to ventricular dilation, impaired compliance and progressive dysfunction (McDonagh et al., 2022; Travers et al., 2016). Fibrosis, however, is not produced by a uniform fibroblast population; cardiac fibroblasts occupy dynamic, injury-dependent states (Tallquist and Molkentin, 2017; Henderson et al., 2020; Tallquist, 2020; Soliman et al., 2020).

Single-cell and single-nucleus studies have revealed marked cardiac stromal heterogeneity (Farbehi et al., 2019; Chaffin et al., 2022). Activated fibroblasts can be sparse in non-failing myocardium but enriched in cardiomyopathy (Chaffin et al., 2022), and MEOX1 has been identified as a transcriptional regulator associated with fibroblast activation (Alexanian et al., 2021). It remains uncertain which human DCM fibroblast state is most reproducibly enriched across cohorts and whether this disease association is stronger than that of a conventional ACTA2-high state.

We therefore reanalyzed two independent human cardiac datasets with donors as the unit of inference. We tested a focused hypothesis: DCM is associated with enrichment of a MEOX1+FAP+, relatively ACTA2-low ECM-remodeling fibroblast state. DDAH1 was evaluated separately as a cross-cohort candidate. The study was designed as a rigorous hypothesis-generating analysis rather than a mechanistic demonstration.

## Methods

### Study design and data sources

This prespecified discovery-and-external-validation analysis used two independent publicly available human cardiac single-nucleus RNA-sequencing cohorts. GSE183852 was the discovery cohort (Koenig et al., 2022) and contained left-ventricular nuclei from 25 non-failing donor and 13 DCM hearts. SCP1303 was the external cohort (Chaffin et al., 2022) and contained 16 normal, 11 DCM and 15 hypertrophic-cardiomyopathy hearts; the prespecified primary validation contrast was DCM versus normal. HCM samples were not included in primary group testing.

### Preprocessing and fibroblast reclustering

Quality-controlled nuclei and source annotations were retained from the public resources. Discovery-cohort fibroblasts were reprocessed in Seurat (Butler et al., 2018; Stuart et al., 2019; Hao et al., 2021) using log normalization (NormalizeData), 2,500 variable features, scaling of variable features, 30 principal components, a shared-nearest-neighbor graph based on principal components 1-25, Louvain graph clustering at resolution 0.5, and UMAP based on principal components 1-25. The random seed for clustering and UMAP was 20260813. The discovery analysis included 49,124 fibroblast nuclei. Harmony embeddings from the source analysis were used only for exploratory visualization and were not used to construct the reported reclustered state or for donor-level inference (Korsunsky et al., 2019). External-cohort author annotations were retained to avoid redefining a favorable validation state. Analyses used R 4.6.1 and Python 3.11; the retained Python manifest records NumPy 2.4.6 and pandas 2.3.3. The exact Seurat package version was not preserved in the original session record and is therefore not inferred retrospectively. Complete scripts and fixed parameters are supplied in Supplementary Code 1.

### State definition

The discovery state was defined by the combined pattern of high MEOX1 and FAP expression with lower ACTA2 expression relative to the discrete ACTA2-high fibroblast state. ACTA2-low denotes a relative state property and does not imply absent ACTA2 expression or lack of overall ACTA2 induction in DCM. DDAH1 was excluded from the state-defining panel and evaluated separately as a priority candidate. Unbiased cluster markers were calculated by comparing cluster 2 with all remaining fibroblasts using normalized expression, a minimum detection fraction of 0.10 and a log_2_-fold-change threshold of 0.25. To assess clustering sensitivity, default cluster-2 cells were mapped to their modal counterparts across SCT graph resolutions 0.3, 0.5, 0.8 and 1.0; purity, recovery and Jaccard overlap were calculated, and donor-level disease enrichment was reassessed for the higher-resolution counterparts (Hafemeister and Satija, 2019).

### Donor-level state abundance

For each donor, state abundance was calculated as the number of nuclei assigned to the target state divided by the total number of fibroblast nuclei. The discovery endpoint was reclustered cluster 2. External support used the author-annotated FB-ActFB population with concordant MEOX1/FAP expression. Continuous donor proportions, rather than cell counts or binary patient labels, were used for inference.

### Bootstrap and leave-one-donor-out analyses

Uncertainty in the between-group mean abundance difference was quantified by nonparametric bootstrap resampling of donors within groups for 10,000 iterations using the fixed random seed 20260825. The 2.5th and 97.5th percentiles formed the 95% confidence interval. Leave-one-donor-out analyses repeated the group contrast after sequential omission of each donor and recorded the effect direction and Wilcoxon *P* value.

### Covariate sensitivity analyses

Heteroscedasticity-consistent HC3 linear models estimated the DCM abundance coefficient. GSE183852 models were unadjusted, sex adjusted, and adjusted for sex plus ordinal age tertile. Continuous age was unavailable. SCP1303 models were unadjusted and sex adjusted; usable age information was unavailable. Reliable within-DCM etiology fields were not available in either retained dataset, so etiology was not imputed or inferred.

### Donor-pseudobulk differential expression

To account for biological-replicate variation and avoid treating nuclei as independent replicates, raw fibroblast counts were summed within each donor (Squair et al., 2021; Soneson and Robinson, 2018; Crowell et al., 2020). Genes with at least one count per million in at least three donors were retained. Expression was represented as log_2_(CPM+0.5). For each gene, an ordinary least-squares model with HC3 robust covariance estimated the DCM coefficient after adjustment for ordinal age tertile and sex. Benjamini-Hochberg false-discovery-rate correction was applied across genes (Benjamini and Hochberg, 1995). This analysis captures disease-associated fibroblast expression and is not interpreted as cluster-specific causality.

### ECM score sensitivity analysis

A prespecified ECM module included COL1A1, COL1A2, COL3A1, COL5A1, COL5A2, FN1, POSTN, DCN, LUM, TGFBI, CTGF, MMP2 and TIMP1. For the pseudobulk score, raw counts were summed across fibroblasts within each donor, converted to counts per million, transformed as log (1 + CPM), and averaged across detected module genes with equal donor weighting. For the cell-derived score, each nucleus was library-size normalized to 10,000 counts, transformed as log (1 + normalized count), averaged across detected ECM genes, and then averaged within donor. Cross-method agreement was assessed with Spearman correlation. Method-specific DCM-versus-donor comparisons used two-sided Mann–Whitney tests.

### DDAH1 prioritization

DDAH1 was evaluated by cross-cohort detection, donor-pseudobulk disease association and within-state ECM association. A nucleus was classified as DDAH1-positive when at least one raw DDAH1 count was detected. Paired positive-versus-negative comparisons were restricted to donors containing both groups within discovery cluster 2; no additional minimum beyond at least one nucleus per group was imposed. Twenty-seven donors were eligible overall, including 13 DCM donors, and two-sided paired Wilcoxon signed-rank tests were used. These observational analyses were used to prioritize DDAH1 for future perturbation studies, not to establish that DDAH1 drives ECM production.

### Statistical analysis and reproducibility

The donor was the primary unit of inference. Tests were two-sided, and exact effect estimates and confidence intervals were emphasized. Benjamini–Hochberg correction was applied separately within the genome-wide donor-pseudobulk gene family and within the fibroblast-state abundance family. Descriptive unbiased marker ranking was reported separately and was not combined with these inferential families. Analyses used R 4.6.1 and Python 3.11 with fixed parameters documented in the additional files.

## Results

### Cross-cohort framework resolves fibroblast-state heterogeneity

GSE183852 provided 25 donor and 13 DCM hearts, and SCP1303 provided 16 normal and 11 DCM hearts for the primary validation comparison (Figure 1A). Reclustering of 49,124 discovery fibroblast nuclei resolved multiple states and substantial between-donor compositional heterogeneity (Figures 1B–D). Accordingly, all primary comparisons were performed on donor-derived proportions or pseudobulk expression rather than treating nuclei as independent replicates.

**FIGURE 1:**
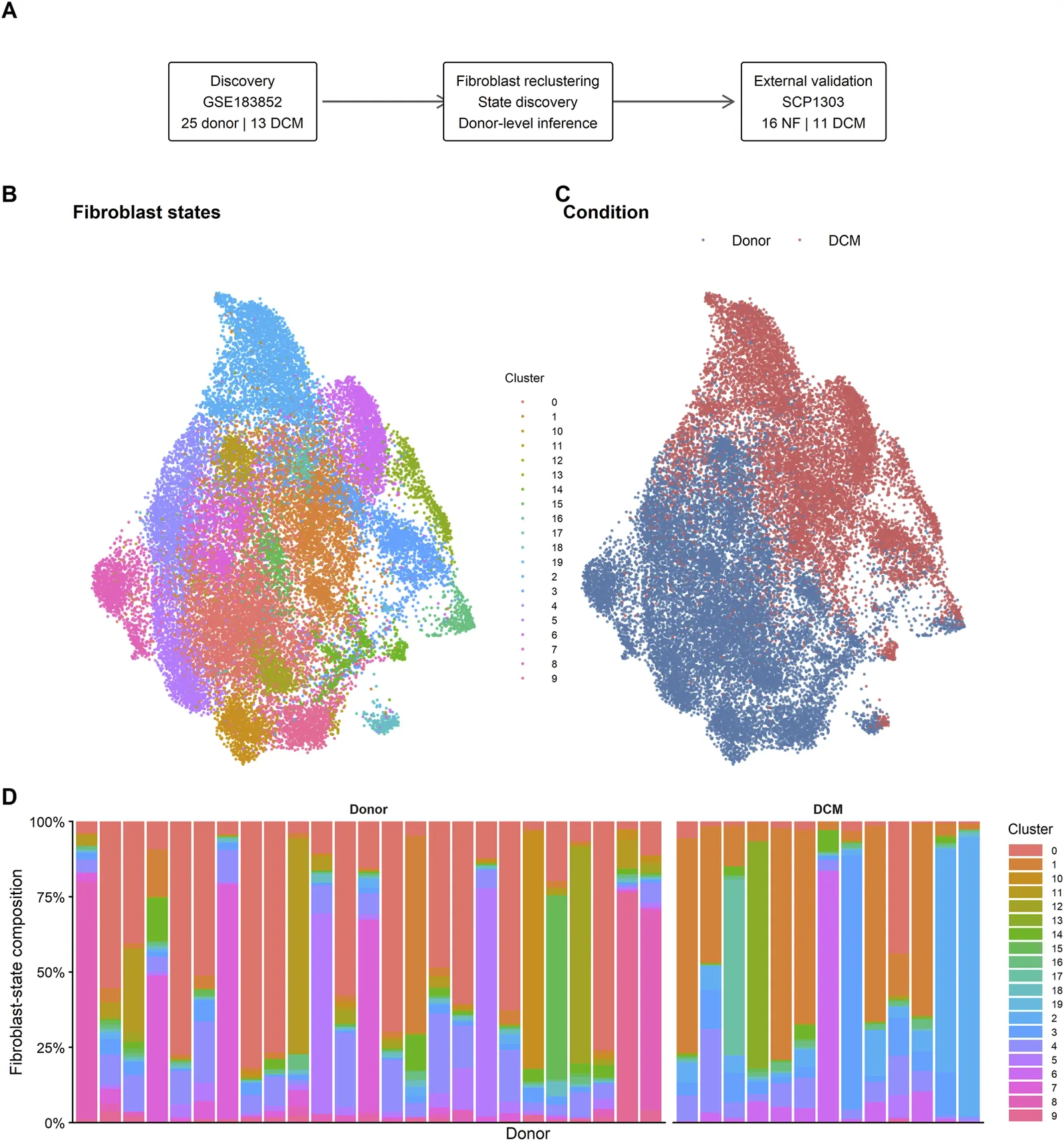
Study design and fibroblast landscape. **(A)** Discovery, state-identification and external-validation workflow. **(B)** GSE183852 fibroblast-state UMAP. **(C)** UMAP by condition. **(D)** Per-donor fibroblast-state composition. Inference used donors rather than nuclei.

### DCM fibroblasts exhibit ECM-program remodeling

The donor-pseudobulk ECM score was higher in DCM (Mann-Whitney *P* = 0.00975; Figure 2A), whereas the cell-derived donor mean showed the same positive direction without a significant group difference (*P* = 0.186). The two summaries were strongly correlated across donors (Spearman *ρ* = 0.891, *P* = 6.62 × 10^−14^; Figure 2B). Donor-pseudobulk models further showed coordinated but non-uniform changes among matrix genes (Figure 2C). Thus, ECM remodeling was supported most strongly at donor-pseudobulk level and was partly dependent on the scoring method.

**FIGURE 2:**
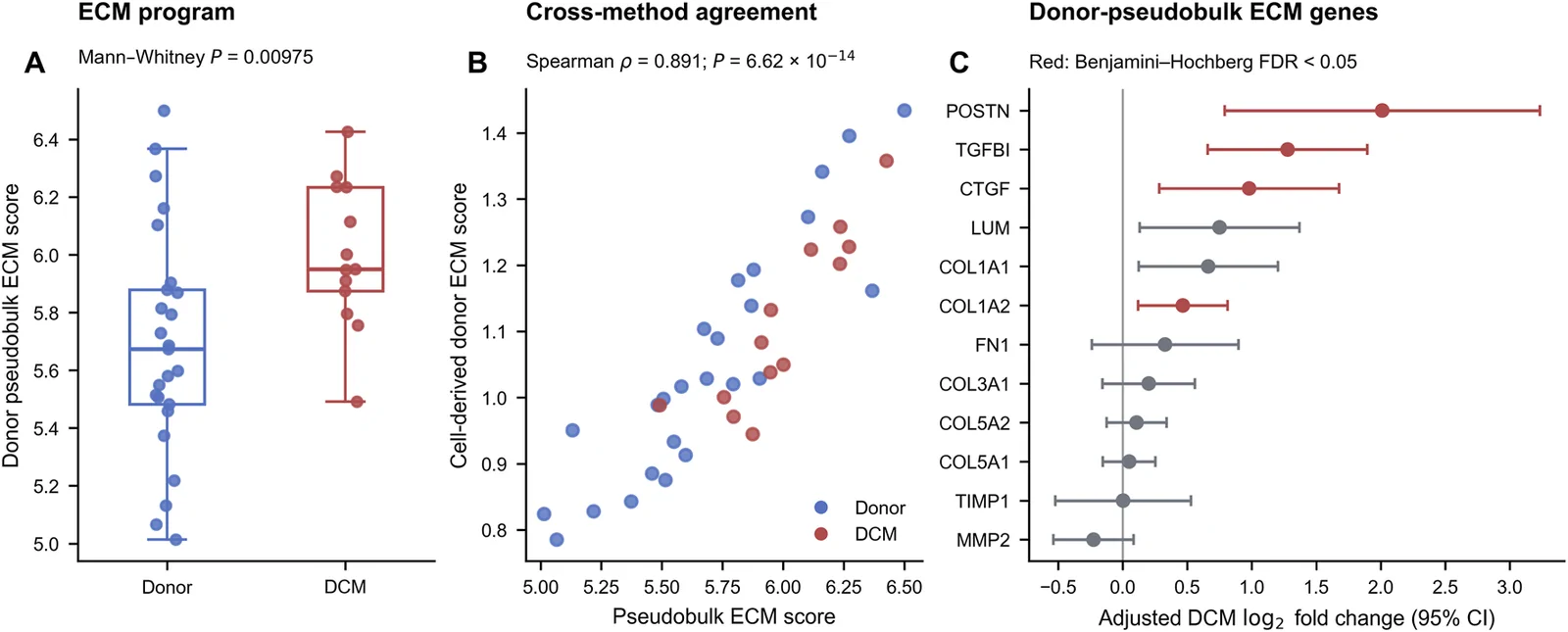
ECM remodeling in DCM fibroblasts. **(A)** Donor-pseudobulk ECM scores using the prespecified Mann–Whitney comparison. **(B)** Agreement between donor-pseudobulk and cell-derived donor ECM summaries. **(C)** Adjusted donor-pseudobulk effect estimates and 95% confidence intervals for representative ECM genes. Each point in panels A and B represents one donor.

### Cluster two defines a non-classical fibroblast state

Cluster two localized MEOX1 and FAP expression with lower ACTA2 than cluster 16, the discrete ACTA2-high state (Figure 3). Cluster two retained ECM-associated genes, including FN1, POSTN, COL1A1 and COL3A1. ACTA2 detection was 14.7% in cluster two compared with 57.3% in cluster 16. In an unbiased cluster-2-versus-rest analysis, the leading detected markers included RFX2, CYR61, FOS, IQCJ-SCHIP1, EGR1, DDAH1, CTGF, EMP1, FAP and MEOX1 (Supplementary Table S7). The combined MEOX1+FAP + ACTA2-low pattern, rather than a single marker, therefore defined the discovery state.

**FIGURE 3:**
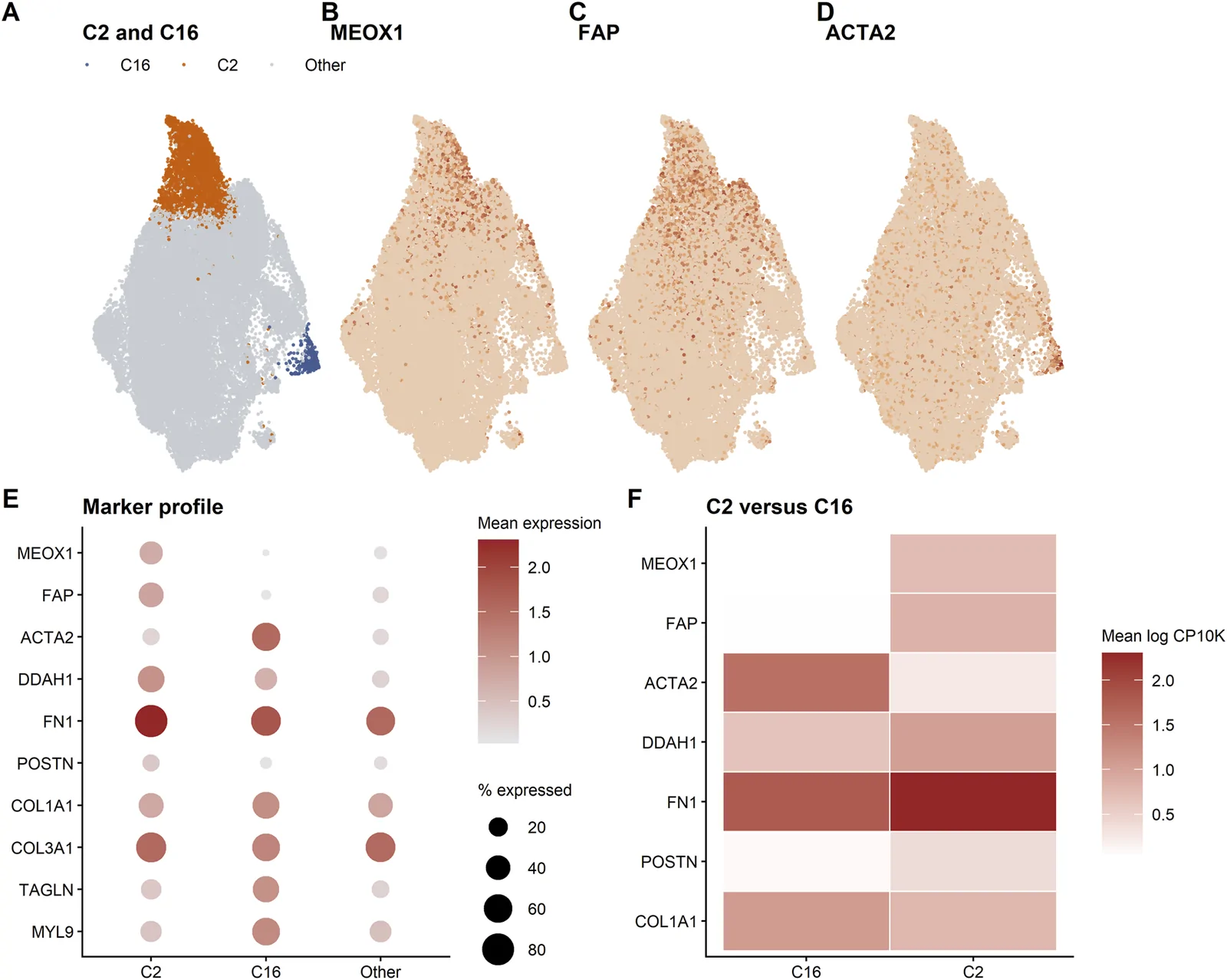
Definition of the MEOX1+FAP + ACTA2-low state. **(A)** Discovery cluster 2, ACTA2-high cluster 16 and other fibroblast states. **(B–D)** MEOX1, FAP and ACTA2 feature plots. **(E)** Marker-expression dot plot. **(F)** Mean-expression heatmap contrasting clusters 2 and 16.

### State enrichment is robust to donor resampling

Cluster two represented a median 5.81% of fibroblasts in DCM and 0.28% in donor hearts (Wilcoxon *P* = 2.83 × 10^−5^; FDR = 1.13 × 10^−4^; Figure 4). The mean DCM-minus-donor difference was 0.165, with a donor-bootstrap 95% confidence interval of 0.035–0.349 (Supplementary Table S1). Sequential omission of each of the 38 donors retained a positive difference (range 0.102–0.179; Supplementary Figure S2). Resolution sensitivity showed that the default state merged into broader compartments at resolutions 0.3–0.5 but retained a disease-enriched modal counterpart at resolutions 0.8 and 1.0 (both Mann–Whitney *P* < 2 × 10^−6^; Supplementary Figure S1; Supplementary Tables S5–S6). The ACTA2-high cluster 16 showed a smaller abundance difference (median 1.43% versus 0.72%; *P* = 0.0605).

**FIGURE 4:**
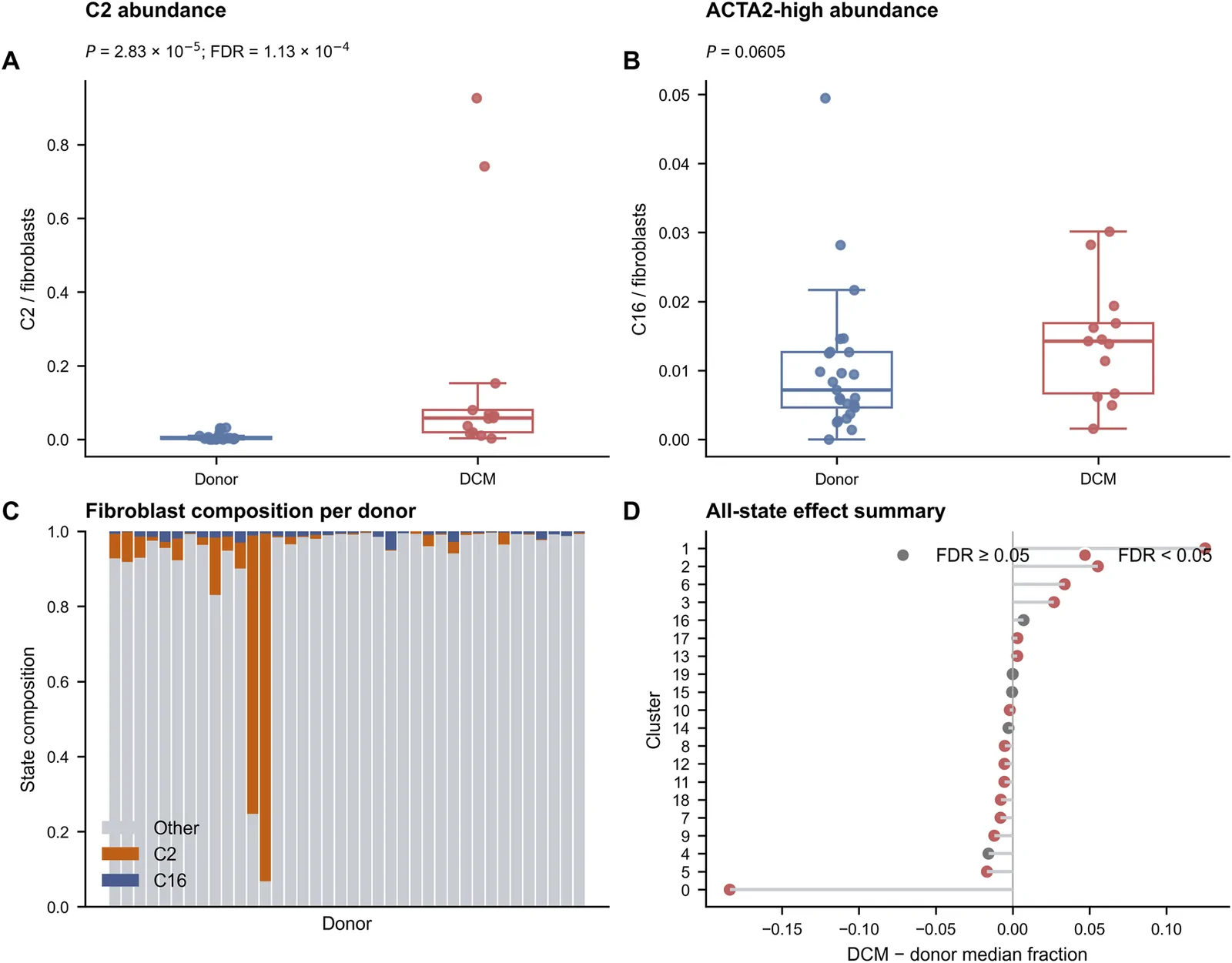
Donor-level enrichment of fibroblast states. **(A)** Cluster two abundance. **(B)** ACTA2-high cluster 16 abundance. **(C)** Per-donor composition. **(D)** DCM-minus-donor abundance differences across states. Nuclei were not treated as independent replicates.

### Covariate models preserve the discovery effect magnitude

The unadjusted HC3 model estimated a DCM coefficient of 0.165 (95% CI −0.004 to 0.334; *P* = 0.0556). Adjustment for sex yielded 0.166 (95% CI −0.007 to 0.338; *P* = 0.0600), and adjustment for age tertile and sex yielded 0.184 (95% CI −0.005 to 0.373; *P* = 0.0560). These analyses preserved the effect magnitude but did not justify a claim of covariate-independent statistical significance (Supplementary Table S2).

### External validation supports a concordant activated state

External validation in the independent SCP1303 patient cohort showed that author-annotated FB-ActFB abundance, calculated as FB-ActFB nuclei divided by all fibroblast nuclei for each donor, was higher in DCM than normal hearts (median 24.11% versus 5.16%; *P* = 1.27 × 10^−5^; Figure 5A; Supplementary Table S4). The mean difference was 0.192 (bootstrap 95% CI 0.144–0.256), and all 27 leave-one-donor-out estimates remained positive (range 0.174–0.203). The sex-adjusted DCM coefficient was 0.199 (95% CI 0.120–0.277; *P* = 6.61 × 10^−7^). The external state showed concordant MEOX1 and FAP expression; ACTA2 was not available in the retained external marker summary. Accordingly, this result validates a concordant activation program and abundance shift, not the complete three-marker phenotype or identical cellular identity.

**FIGURE 5:**
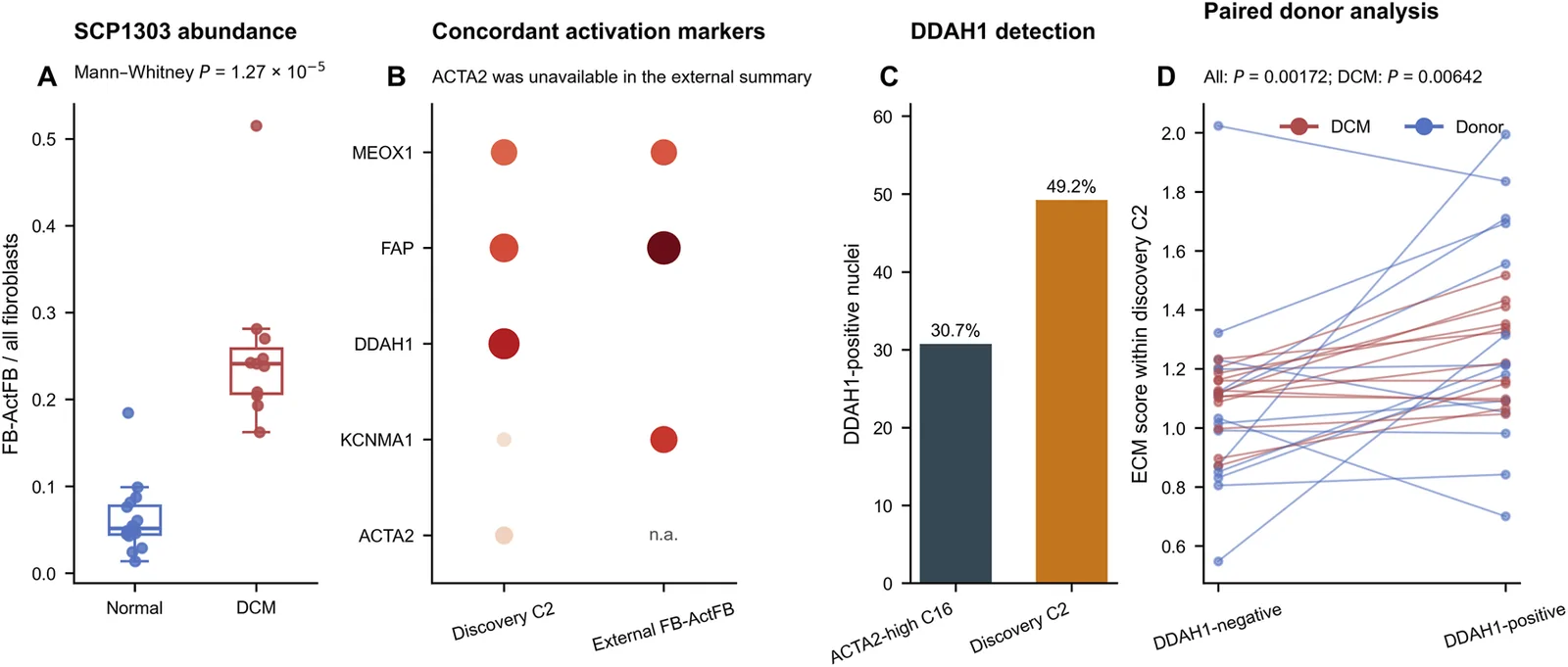
External validation and DDAH1 prioritization. **(A)** SCP1303 FB-ActFB abundance using FB-ActFB nuclei divided by all fibroblast nuclei per donor. **(B)** Concordant activation-marker expression; ACTA2 was unavailable in the retained external summary. **(C)** DDAH1 detection in discovery states. **(D)** Donor-paired ECM-score comparison by DDAH1 detection. DDAH1 is an association-based candidate.

### Pseudobulk analysis prioritizes DDAH1 without establishing causality

Age-tertile- and sex-adjusted donor-pseudobulk models identified DDAH1 (adjusted log_2_ fold change 2.01; FDR = 1.39 × 10^−8^), MEOX1 (2.29; FDR = 3.44 × 10^−6^) and FAP (1.89; FDR = 3.35 × 10^−5^) as increased in DCM fibroblasts. POSTN, TGFBI, CTGF and COL1A2 also passed FDR correction, whereas several ECM genes did not. ACTA2 showed a modest overall increase (adjusted log_2_ fold change 0.54; FDR = 0.017), reinforcing that ACTA2-low is a relative cluster phenotype rather than a whole-disease claim (Supplementary Table S3). Within discovery cluster 2, DDAH1-positive nuclei showed higher ECM scores than DDAH1-negative nuclei in paired donor summaries (Figure 5). Collectively, these findings prioritize DDAH1 as an associated candidate but do not demonstrate functional regulation.

## Discussion

This cross-cohort study supports reproducible remodeling of the fibroblast-state landscape in human DCM. The principal finding is not that ACTA2-high myofibroblasts are absent, but that a MEOX1+FAP+, relatively ACTA2-low discovery state showed disease enrichment and mapped to a concordant activated-fibroblast program in an independent cohort. The conclusion is supported by donor-level inference, bootstrap intervals, leave-one-donor-out stability, covariate sensitivity analysis, resolution sensitivity and external-cohort concordance.

The findings refine the conventional emphasis on a single ACTA2-high myofibroblast phenotype. Cardiac fibroblasts occupy context-dependent transcriptional states (Tallquist and Molkentin, 2017; Tallquist, 2020; Soliman et al., 2020; Patrick et al., 2024), and MEOX1 has been implicated in fibroblast activation (Alexanian et al., 2021). Here, the disease-associated state retained ECM-remodeling features while remaining distinguishable from a discrete contractile ACTA2-high state. Importantly, whole-fibroblast pseudobulk ACTA2 was modestly increased in DCM; therefore, the present data do not refute canonical myofibroblast biology. This interpretation is consistent with prior work on extracellular-matrix remodeling, fibroblast-state modulation, and molecular regulators of cardiac fibrosis (Louzao-Martinez et al., 2016; Gil-Cayuela et al., 2016; Reichardt et al., 2021; Shi and Furtado, 2021; Villalobos et al., 2019; Eguchi et al., 2021; Andenæs et al., 2018).

The external data strengthened the observation but also defined its boundary. SCP1303 reproduced an activated-fibroblast abundance shift and concordant MEOX1/FAP expression. Because ACTA2 was unavailable in the retained external marker summary and source-study annotations rather than frozen one-to-one label transfer were used, the evidence supports a shared activation program rather than complete replication of the MEOX1+FAP + ACTA2-low phenotype.

DDAH1 emerged as a reproducible candidate through state enrichment, overall fibroblast pseudobulk association and ECM-score association. Prior studies link DDAH1 biology to cardiac hypertrophy and remodeling (Xu et al., 2017; Kopaliani et al., 2021), but the current analysis cannot determine whether fibroblast DDAH1 is causal, compensatory or simply co-expressed with activation. DDAH1 should therefore remain outside the state-definition panel and be tested by perturbation and spatial localization.

This study has limitations. It is a secondary analysis of public data without spatial, protein-level or functional validation. Available covariates were incomplete: GSE183852 provided age tertiles rather than continuous age, SCP1303 lacked usable age information, and reliable DCM etiologic subdivisions were unavailable. ECM significance differed between two scoring summaries despite strong correlation. Whole-fibroblast pseudobulk differences can reflect both changes in fibroblast-state composition and expression changes within states, and therefore cannot establish cluster-specific regulation. Lower clustering resolutions merged the discovery state into broader compartments, whereas higher-resolution counterparts retained disease enrichment (Kiselev et al., 2019; Luecken and Theis, 2019). Public-cohort processing and annotation differences precluded exact state transfer, and the analyzed samples may not represent all DCM phenotypes. These limitations preclude mechanistic or universal claims.

Nevertheless, the work provides a technically controlled, hypothesis-generating foundation for prospective studies. Multiplex tissue imaging should test the spatial coexistence of MEOX1, FAP, ACTA2 and DDAH1, while fibroblast perturbation should determine whether DDAH1 alters collagen synthesis, matrix organization or fibroblast state transitions.

## Conclusion

Across two public human cardiac cohorts, DCM was associated with a reproducible MEOX1+FAP+, relatively ACTA2-low fibroblast state and ECM-program remodeling. DDAH1 is a prioritized state-associated candidate requiring spatial and functional validation. These data support a focused experimental hypothesis but do not establish causality.

## Data Availability

The public datasets analyzed in this study are available from the Gene Expression Omnibus under accession GSE183852 and the Broad Institute Single Cell Portal under study accession SCP1303. The derived supplementary materials, source data, and analysis code are available in Zenodo at https://doi.org/10.5281/zenodo.23282470 and are also provided with the article as Supplementary Material. Further inquiries can be directed to the corresponding author.
