# Supplementary material for: Cross-cohort single-nucleus transcriptomic identification of a MEOX1+FAP+ACTA2-low extracellular matrix-remodeling fibroblast state in dilated cardiomyopathy
Source: Front Genet. 2026 Oct 19;17:1987893. doi: 10.3389/fgene.2026.1987893 (PMC13724366; doi:10.3389/fgene.2026.1987893)
Supplement: Supplementary file 1 [file Table1.docx]

**Supplementary Material**

Cross-cohort single-nucleus transcriptomic identification of a MEOX1+FAP+ACTA2-low extracellular matrix-remodeling fibroblast state in dilated cardiomyopathy

# **Supplementary methods**

## **Discovery fibroblast reclustering**

Fibroblasts were reprocessed with Seurat log normalization, 2,500 variable features, scaling of variable features, 30 principal components, shared-nearest-neighbor graph construction using principal components 1-25, clustering at resolution 0.5, and UMAP using principal components 1-25. The random seed was 20260813. Harmony was not used to construct the reported reclustered state. Analyses used R 4.6.1 and Python 3.11; the retained Python manifest records NumPy 2.4.6 and pandas 2.3.3. The exact Seurat package version was not preserved and is explicitly reported as unavailable rather than inferred.

## **Unbiased marker analysis**

Positive cluster markers were identified by comparing cluster 2 with all other fibroblasts using normalized expression, a minimum detection fraction of 0.10 and a log_2_-fold-change threshold of 0.25. The full ranking is supplied in Supplementary Data Sheet 1; the leading markers detected in more than 20% of cluster-2 nuclei are summarized in Supplementary Table S7.

## **Resolution sensitivity**

Default cluster-2 cells were mapped to their modal counterparts at SCT graph resolutions 0.3, 0.5, 0.8 and 1.0. Purity was the fraction of counterpart cells belonging to default cluster 2; recovery was the fraction of default cluster-2 cells captured by the counterpart; Jaccard overlap used the union of both cell sets. Donor-level disease enrichment was reassessed for the higher-resolution counterparts.

## **Bootstrap and leave-one-donor-out analyses**

Donors were resampled within disease groups for 10,000 nonparametric bootstrap iterations using seed 20260825. Leave-one-donor-out analyses sequentially omitted each donor and repeated the disease contrast.

## **ECM score sensitivity**

The pseudobulk ECM score used donor-summed counts, CPM normalization, log(1 + CPM) transformation and the mean across detected module genes. The cell-derived score used per-nucleus library normalization to 10,000 counts, log(1 + normalized count), the mean across detected ECM genes, and a final donor mean. Both group comparisons used two-sided Mann–Whitney tests.

## **Paired DDAH1 analysis**

A nucleus was DDAH1-positive when at least one raw DDAH1 count was detected. Donors were eligible when discovery cluster 2 contained at least one DDAH1-positive and one DDAH1-negative nucleus. Twenty-seven donors, including 13 DCM donors, were eligible. Positive and negative donor means were compared with two-sided paired Wilcoxon signed-rank tests.

## **False-discovery-rate families**

Benjamini–Hochberg correction was applied separately across the genome-wide donor-pseudobulk gene family and across fibroblast-state abundance comparisons. Unbiased marker rankings were treated as descriptive and reported separately.

## **Supplementary Figure S1. Resolution sensitivity and unbiased marker analysis**

**(A)** Purity, recovery and Jaccard overlap between default cluster 2 and its modal counterpart across SCT graph resolutions. Lower resolutions merge the state into broader compartments; resolutions 0.8 and 1.0 retain a partially overlapping counterpart. **(B)** Donor-level disease enrichment of the modal counterparts at resolutions 0.8 and 1.0. **(C)** Leading unbiased cluster-2 markers among genes detected in more than 20% of cluster-2 nuclei.

## **Supplementary Figure S2. Donor-level robustness analyses**

**(A)** Donor-bootstrap estimates and 95% confidence intervals. **(B)** Leave-one-donor-out abundance differences. **(C)** HC3 covariate-model estimates. **(D)** ECM-score method sensitivity. All effects are DCM minus control.

## **Supplementary Table S1. Bootstrap estimates**

| **Cohort** | **Cases, n** | **Controls, n** | **Mean difference** | **95% CI, lower** | **95% CI, upper** |
| --- | --- | --- | --- | --- | --- |
| GSE183852 | 13 | 25 | 0.1651 | 0.0352 | 0.3487 |
| SCP1303 | 11 | 16 | 0.1925 | 0.1442 | 0.2558 |

## **Supplementary Table S2. Covariate sensitivity models**

| **Cohort** | **Model** | **Donors, n** | **DCM coefficient** | **95% CI, lower** | **95% CI, upper** | **DCM *P* value** |
| --- | --- | --- | --- | --- | --- | --- |
| GSE183852 | unadjusted | 38 | 0.1651 | -0.0040 | 0.3342 | 0.0556 |
| GSE183852 | sex_adjusted | 38 | 0.1656 | -0.0070 | 0.3383 | 0.06 |
| GSE183852 | age_tertile_and_sex_adjusted | 38 | 0.1843 | -0.0047 | 0.3733 | 0.056 |
| SCP1303 | unadjusted | 27 | 0.1925 | 0.1315 | 0.2534 | 6.19e-10 |
| SCP1303 | sex_adjusted | 27 | 0.1988 | 0.1205 | 0.2772 | 6.61e-07 |

## **Supplementary Table S3. Priority-gene donor-pseudobulk results**

| **Gene** | **Adjusted DCM log_2_ fold change** | **95% CI, lower** | **95% CI, upper** | ***P* value** | **FDR** |
| --- | --- | --- | --- | --- | --- |
| DDAH1 | 2.008 | 1.388 | 2.628 | 2.21e-10 | 1.39e-08 |
| MEOX1 | 2.286 | 1.436 | 3.136 | 1.35e-07 | 3.44e-06 |
| FAP | 1.887 | 1.109 | 2.666 | 2.02e-06 | 3.35e-05 |
| DCN | -0.924 | -1.333 | -0.514 | 9.77e-06 | 0.000128 |
| TGFBI | 1.278 | 0.658 | 1.897 | 5.36e-05 | 0.000522 |
| POSTN | 2.012 | 0.790 | 3.234 | 0.00125 | 0.00665 |
| ACTA2 | 0.535 | 0.171 | 0.900 | 0.00403 | 0.017 |
| CTGF | 0.979 | 0.282 | 1.677 | 0.00592 | 0.023 |
| COL1A2 | 0.465 | 0.119 | 0.812 | 0.00848 | 0.0307 |
| COL1A1 | 0.663 | 0.123 | 1.202 | 0.0161 | 0.0504 |
| LUM | 0.750 | 0.132 | 1.368 | 0.0174 | 0.0536 |
| MMP2 | -0.226 | -0.537 | 0.085 | 0.154 | 0.28 |

## **Supplementary Table S4. SCP1303 donor-level FB-ActFB numerator and denominator**

| **Donor** | **Disease** | **Sex** | **FB-ActFB nuclei** | **All fibroblast nuclei** | **FB-ActFB fraction** |
| --- | --- | --- | --- | --- | --- |
| P1290 | dilated cardiomyopathy | male | 262 | 971 | 0.26982 |
| P1300 | dilated cardiomyopathy | female | 728 | 3558 | 0.20461 |
| P1304 | dilated cardiomyopathy | female | 4533 | 8807 | 0.51470 |
| P1358 | dilated cardiomyopathy | male | 1231 | 6383 | 0.19286 |
| P1371 | dilated cardiomyopathy | male | 596 | 3674 | 0.16222 |
| P1430 | dilated cardiomyopathy | male | 1007 | 3584 | 0.28097 |
| P1437 | dilated cardiomyopathy | female | 1873 | 7737 | 0.24208 |
| P1472 | dilated cardiomyopathy | male | 1203 | 5771 | 0.20846 |
| P1504 | dilated cardiomyopathy | male | 540 | 2187 | 0.24691 |
| P1606 | dilated cardiomyopathy | female | 772 | 3238 | 0.23842 |
| P1617 | dilated cardiomyopathy | male | 1313 | 5446 | 0.24109 |
| P1515 | normal | female | 234 | 3869 | 0.06048 |
| P1516 | normal | female | 182 | 1838 | 0.09902 |
| P1539 | normal | female | 127 | 2815 | 0.04512 |
| P1540 | normal | female | 62 | 1440 | 0.04306 |
| P1547 | normal | female | 115 | 1315 | 0.08745 |
| P1549 | normal | male | 155 | 3367 | 0.04604 |
| P1558 | normal | female | 119 | 2321 | 0.05127 |
| P1561 | normal | male | 174 | 3355 | 0.05186 |
| P1582 | normal | female | 141 | 2888 | 0.04882 |
| P1600 | normal | female | 41 | 1684 | 0.02435 |
| P1603 | normal | female | 160 | 2945 | 0.05433 |
| P1610 | normal | female | 219 | 2689 | 0.08144 |
| P1622 | normal | male | 164 | 2146 | 0.07642 |
| P1678 | normal | male | 44 | 1526 | 0.02883 |
| P1702 | normal | male | 18 | 1333 | 0.01350 |
| P1718 | normal | male | 496 | 2688 | 0.18452 |

## **Supplementary Table S5. Mapping of default cluster 2 across clustering resolutions**

| **Resolution** | **Counterpart cluster** | **Purity** | **Recovery** | **Jaccard overlap** |
| --- | --- | --- | --- | --- |
| 0.3 | 0.0 | 0.1013 | 0.7958 | 0.0987 |
| 0.5 | 0.0 | 0.0957 | 0.7034 | 0.0920 |
| 0.8 | 5.0 | 0.2343 | 0.7077 | 0.2136 |
| 1.0 | 5.0 | 0.2291 | 0.6329 | 0.2022 |

## **Supplementary Table S6. Donor-level abundance of modal counterparts across clustering resolutions**

| **Donor** | **Condition** | **Resolution** | **Counterpart cluster** | **Counterpart fraction** |
| --- | --- | --- | --- | --- |
| H_ZC-11-292 | Donor | 0.3 | 0 | 0.94050 |
| H_ZC-LVAD | DCM | 0.3 | 0 | 0.95053 |
| TWCM-10^-5^ | DCM | 0.3 | 0 | 0.91411 |
| TWCM-10^-68^ | Donor | 0.3 | 0 | 0.94891 |
| TWCM-11-103 | Donor | 0.3 | 0 | 0.86548 |
| TWCM-11-104 | Donor | 0.3 | 0 | 0.97652 |
| TWCM-11-192 | Donor | 0.3 | 0 | 0.98536 |
| TWCM-11-256 | Donor | 0.3 | 0 | 0.95393 |
| TWCM-11-264 | Donor | 0.3 | 0 | 0.93241 |
| TWCM-11-3 | DCM | 0.3 | 0 | 0.87535 |
| TWCM-11-41 | Donor | 0.3 | 0 | 0.96561 |
| TWCM-11-42 | Donor | 0.3 | 0 | 0.98136 |
| TWCM-11-74 | Donor | 0.3 | 0 | 0.75489 |
| TWCM-11-78 | Donor | 0.3 | 0 | 0.97026 |
| TWCM-11-82 | Donor | 0.3 | 0 | 0.98442 |
| TWCM-11-93 | DCM | 0.3 | 0 | 0.57374 |
| TWCM-13-1 | Donor | 0.3 | 0 | 0.95579 |
| TWCM-13-101 | Donor | 0.3 | 0 | 0.97598 |
| TWCM-13-102 | DCM | 0.3 | 0 | 0.94952 |
| TWCM-13-104 | Donor | 0.3 | 0 | 0.92484 |
| TWCM-13-132 | Donor | 0.3 | 0 | 0.96005 |
| TWCM-13-152 | Donor | 0.3 | 0 | 0.99073 |
| TWCM-13-168 | Donor | 0.3 | 0 | 0.99322 |
| TWCM-13-17 | DCM | 0.3 | 0 | 0.93838 |
| TWCM-13-181 | DCM | 0.3 | 0 | 0.95509 |
| TWCM-13-192 | Donor | 0.3 | 0 | 0.97952 |
| TWCM-13-198 | Donor | 0.3 | 0 | 0.92233 |
| TWCM-13-208 | DCM | 0.3 | 0 | 0.59676 |
| TWCM-13-235 | Donor | 0.3 | 0 | 0.96902 |
| TWCM-13-285 | DCM | 0.3 | 0 | 0.95040 |
| TWCM-13-36 | Donor | 0.3 | 0 | 0.95539 |
| TWCM-13-47 | DCM | 0.3 | 0 | 0.87753 |
| TWCM-13-80 | Donor | 0.3 | 0 | 0.98607 |
| TWCM-13-84 | DCM | 0.3 | 0 | 0.93085 |
| TWCM-13-96 | Donor | 0.3 | 0 | 0.92285 |
| TWCM-14-173 | Donor | 0.3 | 0 | 0.97952 |
| TWCM-LVAD2 | DCM | 0.3 | 0 | 0.81383 |
| TWCM-LVAD3 | DCM | 0.3 | 0 | 0.74082 |
| H_ZC-11-292 | Donor | 0.5 | 0 | 0.88320 |
| H_ZC-LVAD | DCM | 0.5 | 0 | 0.92218 |
| TWCM-10^-5^ | DCM | 0.5 | 0 | 0.90031 |
| TWCM-10^-68^ | Donor | 0.5 | 0 | 0.91423 |
| TWCM-11-103 | Donor | 0.5 | 0 | 0.75912 |
| TWCM-11-104 | Donor | 0.5 | 0 | 0.85530 |
| TWCM-11-192 | Donor | 0.5 | 0 | 0.95816 |
| TWCM-11-256 | Donor | 0.5 | 0 | 0.92898 |
| TWCM-11-264 | Donor | 0.5 | 0 | 0.88128 |
| TWCM-11-3 | DCM | 0.5 | 0 | 0.84488 |
| TWCM-11-41 | Donor | 0.5 | 0 | 0.94085 |
| TWCM-11-42 | Donor | 0.5 | 0 | 0.92943 |
| TWCM-11-74 | Donor | 0.5 | 0 | 0.47296 |
| TWCM-11-78 | Donor | 0.5 | 0 | 0.90616 |
| TWCM-11-82 | Donor | 0.5 | 0 | 0.94545 |
| TWCM-11-93 | DCM | 0.5 | 0 | 0.54646 |
| TWCM-13-1 | Donor | 0.5 | 0 | 0.93569 |
| TWCM-13-101 | Donor | 0.5 | 0 | 0.94595 |
| TWCM-13-102 | DCM | 0.5 | 0 | 0.93615 |
| TWCM-13-104 | Donor | 0.5 | 0 | 0.75678 |
| TWCM-13-132 | Donor | 0.5 | 0 | 0.91079 |
| TWCM-13-152 | Donor | 0.5 | 0 | 0.95088 |
| TWCM-13-168 | Donor | 0.5 | 0 | 0.94701 |
| TWCM-13-17 | DCM | 0.5 | 0 | 0.90317 |
| TWCM-13-181 | DCM | 0.5 | 0 | 0.88247 |
| TWCM-13-192 | Donor | 0.5 | 0 | 0.94759 |
| TWCM-13-198 | Donor | 0.5 | 0 | 0.85802 |
| TWCM-13-208 | DCM | 0.5 | 0 | 0.58234 |
| TWCM-13-235 | Donor | 0.5 | 0 | 0.93632 |
| TWCM-13-285 | DCM | 0.5 | 0 | 0.92659 |
| TWCM-13-36 | Donor | 0.5 | 0 | 0.90249 |
| TWCM-13-47 | DCM | 0.5 | 0 | 0.84542 |
| TWCM-13-80 | Donor | 0.5 | 0 | 0.94011 |
| TWCM-13-84 | DCM | 0.5 | 0 | 0.91844 |
| TWCM-13-96 | Donor | 0.5 | 0 | 0.84375 |
| TWCM-14-173 | Donor | 0.5 | 0 | 0.94227 |
| TWCM-LVAD2 | DCM | 0.5 | 0 | 0.77584 |
| TWCM-LVAD3 | DCM | 0.5 | 0 | 0.63710 |
| H_ZC-11-292 | Donor | 0.8 | 5 | 0.05675 |
| H_ZC-LVAD | DCM | 0.8 | 5 | 0.71984 |
| TWCM-10^-5^ | DCM | 0.8 | 5 | 0.70092 |
| TWCM-10^-68^ | Donor | 0.8 | 5 | 0.18248 |
| TWCM-11-103 | Donor | 0.8 | 5 | 0.07925 |
| TWCM-11-104 | Donor | 0.8 | 5 | 0.33182 |
| TWCM-11-192 | Donor | 0.8 | 5 | 0.06172 |
| TWCM-11-256 | Donor | 0.8 | 5 | 0.10173 |
| TWCM-11-264 | Donor | 0.8 | 5 | 0.05893 |
| TWCM-11-3 | DCM | 0.8 | 5 | 0.76593 |
| TWCM-11-41 | Donor | 0.8 | 5 | 0.03576 |
| TWCM-11-42 | Donor | 0.8 | 5 | 0.08389 |
| TWCM-11-74 | Donor | 0.8 | 5 | 0.14154 |
| TWCM-11-78 | Donor | 0.8 | 5 | 0.05572 |
| TWCM-11-82 | Donor | 0.8 | 5 | 0.04675 |
| TWCM-11-93 | DCM | 0.8 | 5 | 0.49872 |
| TWCM-13-1 | Donor | 0.8 | 5 | 0.41881 |
| TWCM-13-101 | Donor | 0.8 | 5 | 0.05706 |
| TWCM-13-102 | DCM | 0.8 | 5 | 0.78396 |
| TWCM-13-104 | Donor | 0.8 | 5 | 0.63674 |
| TWCM-13-132 | Donor | 0.8 | 5 | 0.11718 |
| TWCM-13-152 | Donor | 0.8 | 5 | 0.04356 |
| TWCM-13-168 | Donor | 0.8 | 5 | 0.04128 |
| TWCM-13-17 | DCM | 0.8 | 5 | 0.75352 |
| TWCM-13-181 | DCM | 0.8 | 5 | 0.82590 |
| TWCM-13-192 | Donor | 0.8 | 5 | 0.24578 |
| TWCM-13-198 | Donor | 0.8 | 5 | 0.00514 |
| TWCM-13-208 | DCM | 0.8 | 5 | 0.41045 |
| TWCM-13-235 | Donor | 0.8 | 5 | 0.09294 |
| TWCM-13-285 | DCM | 0.8 | 5 | 0.84821 |
| TWCM-13-36 | Donor | 0.8 | 5 | 0.04525 |
| TWCM-13-47 | DCM | 0.8 | 5 | 0.34007 |
| TWCM-13-80 | Donor | 0.8 | 5 | 0.03203 |
| TWCM-13-84 | DCM | 0.8 | 5 | 0.84397 |
| TWCM-13-96 | Donor | 0.8 | 5 | 0.01904 |
| TWCM-14-173 | Donor | 0.8 | 5 | 0.02048 |
| TWCM-LVAD2 | DCM | 0.8 | 5 | 0.73176 |
| TWCM-LVAD3 | DCM | 0.8 | 5 | 0.64497 |
| H_ZC-11-292 | Donor | 1.0 | 5 | 0.03747 |
| H_ZC-LVAD | DCM | 1.0 | 5 | 0.63091 |
| TWCM-10^-5^ | DCM | 1.0 | 5 | 0.56595 |
| TWCM-10^-68^ | Donor | 1.0 | 5 | 0.14781 |
| TWCM-11-103 | Donor | 1.0 | 5 | 0.03337 |
| TWCM-11-104 | Donor | 1.0 | 5 | 0.44621 |
| TWCM-11-192 | Donor | 1.0 | 5 | 0.04498 |
| TWCM-11-256 | Donor | 1.0 | 5 | 0.06334 |
| TWCM-11-264 | Donor | 1.0 | 5 | 0.04853 |
| TWCM-11-3 | DCM | 1.0 | 5 | 0.70914 |
| TWCM-11-41 | Donor | 1.0 | 5 | 0.05227 |
| TWCM-11-42 | Donor | 1.0 | 5 | 0.04394 |
| TWCM-11-74 | Donor | 1.0 | 5 | 0.13349 |
| TWCM-11-78 | Donor | 1.0 | 5 | 0.03435 |
| TWCM-11-82 | Donor | 1.0 | 5 | 0.02727 |
| TWCM-11-93 | DCM | 1.0 | 5 | 0.48252 |
| TWCM-13-1 | Donor | 1.0 | 5 | 0.32878 |
| TWCM-13-101 | Donor | 1.0 | 5 | 0.02402 |
| TWCM-13-102 | DCM | 1.0 | 5 | 0.76615 |
| TWCM-13-104 | Donor | 1.0 | 5 | 0.57307 |
| TWCM-13-132 | Donor | 1.0 | 5 | 0.08256 |
| TWCM-13-152 | Donor | 1.0 | 5 | 0.02132 |
| TWCM-13-168 | Donor | 1.0 | 5 | 0.03142 |
| TWCM-13-17 | DCM | 1.0 | 5 | 0.69542 |
| TWCM-13-181 | DCM | 1.0 | 5 | 0.81890 |
| TWCM-13-192 | Donor | 1.0 | 5 | 0.16627 |
| TWCM-13-198 | Donor | 1.0 | 5 | 0.00463 |
| TWCM-13-208 | DCM | 1.0 | 5 | 0.37982 |
| TWCM-13-235 | Donor | 1.0 | 5 | 0.08692 |
| TWCM-13-285 | DCM | 1.0 | 5 | 0.80258 |
| TWCM-13-36 | Donor | 1.0 | 5 | 0.02868 |
| TWCM-13-47 | DCM | 1.0 | 5 | 0.29132 |
| TWCM-13-80 | Donor | 1.0 | 5 | 0.03064 |
| TWCM-13-84 | DCM | 1.0 | 5 | 0.81206 |
| TWCM-13-96 | Donor | 1.0 | 5 | 0.01709 |
| TWCM-14-173 | Donor | 1.0 | 5 | 0.01676 |
| TWCM-LVAD2 | DCM | 1.0 | 5 | 0.66641 |
| TWCM-LVAD3 | DCM | 1.0 | 5 | 0.58250 |

## **Supplementary Table S7. Leading unbiased cluster-2 markers**

| **Gene** | **log_2_ fold change** | **Cluster-2 detection** | **Other-state detection** |
| --- | --- | --- | --- |
| LRRFIP1 | 2.088 | 0.725 | 0.322 |
| EMP1 | 2.003 | 0.600 | 0.308 |
| CTGF | 2.013 | 0.585 | 0.210 |
| LMNA | 2.093 | 0.580 | 0.234 |
| DDAH1 | 2.136 | 0.492 | 0.151 |
| CTD-3252C9.4 | 2.257 | 0.468 | 0.137 |
| LINC00152 | 1.990 | 0.468 | 0.181 |
| RFX2 | 2.861 | 0.452 | 0.102 |
| CYR61 | 2.703 | 0.448 | 0.104 |
| CRY1 | 2.030 | 0.438 | 0.133 |
| EGR1 | 2.262 | 0.420 | 0.089 |
| FAP | 1.975 | 0.417 | 0.123 |
| IQCJ-SCHIP1 | 2.298 | 0.398 | 0.097 |
| FAM102B | 1.986 | 0.397 | 0.115 |
| FOS | 2.322 | 0.386 | 0.092 |
